# The Role of MicroRNA-200 in Progression of Human Colorectal and Breast Cancer

**DOI:** 10.1371/journal.pone.0084815

**Published:** 2013-12-20

**Authors:** Linda Bojmar, Elin Karlsson, Sander Ellegård, Hans Olsson, Bergthor Björnsson, Olof Hallböök, Marie Larsson, Olle Stål, Per Sandström

**Affiliations:** 1 Surgery, Department of Clinical and Experimental Medicine, Faculty of Health Sciences, Linköping University, Department of Surgery in Östergötland, County Council of Östergötland, Linköping, Sweden; 2 Oncology, Department of Clinical and Experimental Medicine, Faculty of Health Sciences, Linköping University, Department of Oncology, County Council of Östergötland, Linköping, Sweden; 3 Pathology, Department of Clinical and Experimental Medicine, Faculty of Health Sciences, Linköping University, Department of Pathology in Östergötland, County Council of Östergötland, Linköping, Sweden; 4 Molecular Virology, Department of Clinical and Experimental Medicine, Faculty of Health Sciences, Linköping University, Linköping, Sweden; University General Hospital of Heraklion and Laboratory of Tumor Cell Biology, School of Medicine, University of Crete, Greece

## Abstract

The role of the epithelial-mesenchymal transition (EMT) in cancer has been studied extensively *in vitro*, but involvement of the EMT in tumorigenesis *in vivo* is largely unknown. We investigated the potential of microRNAs as clinical markers and analyzed participation of the EMT-associated microRNA-200–ZEB–E-cadherin pathway in cancer progression. Expression of the microRNA-200 family was quantified by real-time RT-PCR analysis of fresh-frozen and microdissected formalin-fixed paraffin-embedded primary colorectal tumors, normal colon mucosa, and matched liver metastases. MicroRNA expression was validated by *in situ* hybridization and after *in vitro* culture of the malignant cells. To assess EMT as a predictive marker, factors considered relevant in colorectal cancer were investigated in 98 primary breast tumors from a treatment-randomized study. Associations between the studied EMT-markers were found in primary breast tumors and in colorectal liver metastases. MicroRNA-200 expression in epithelial cells was lower in malignant mucosa than in normal mucosa, and was also decreased in metastatic compared to non-metastatic colorectal cancer. Low microRNA-200 expression in colorectal liver metastases was associated with bad prognosis. In breast cancer, low levels of microRNA-200 were related to reduced survival and high expression of microRNA-200 was predictive of benefit from radiotheraphy. MicroRNA-200 was associated with ER positive status, and inversely correlated to HER2 and overactivation of the PI3K/AKT pathway, that was associated with high ZEB1 mRNA expression. Our findings suggest that the stability of microRNAs makes them suitable as clinical markers and that the EMT-related microRNA-200 – ZEB – E-cadherin signaling pathway is connected to established clinical characteristics and can give useful prognostic and treatment-predictive information in progressive breast and colorectal cancers.

## Introduction

It is believed that epithelial-mesenchymal transition (EMT) plays an important role in cancer progression, especially during metastasis [1]. The EMT is a multifaceted process that normally occurs during embryonic development and cellular differentiation [2], and it has been studied extensively *in vitro* in relation to cancer [3-6]. It has been suggested that the EMT has a critical role in the initial spread of primary tumors, displayed in animal models [7,8], and to some extent in human tissues [9,10], although the data obtained *in vivo* is rare.

Several microRNAs have been associated with EMT and metastasis, and the microRNA-200 (mir-200) family is described most frequently in this context [11]. The present study focused on the mir-200s and the targeted transcription factor *ZEB1*. The mir-200s suppress *ZEB1*, which in turn inhibits expression of the well-known EMT marker E-cadherin (*CDH1*), and thereby supports epithelial maintenance [12].

There are five members of the mir-200 family, which are clustered together at two polycistronic sites: mir-200a, mir-200b, and mir-429 are located on chromosome 1p36 [13]; and mir-200c and mir-141 are located on chromosome 12p13. These microRNAs display marked structural similarities, suggesting that they mainly target the same genes. The wide variety of microRNA targets make them interesting as potential contributors to the plasticity of processes involved in EMT and metastasis. 

The aim of the present study was to examine the potential of microRNAs as clinical markers and to elucidate the participation of the mir-200–ZEB–E-cadherin pathway in the progression of human cancers by using patient materials from metastatic colorectal and breast cancer. 

## Materials and Methods

The design of the present study and the presentation of the results comply with the Reporting Recommendations for Tumor Marker Prognostic Studies (REMARK) guidelines [14].

### Patients

#### Breast cancer

Frozen primary tumor tissue was available from 98 postmenopausal women who had stage II breast cancer and were randomized to postoperative radiotherapy (RT) or systemic chemotherapy (cyclophosphamide, methotrexate, and fluorouracil [CMF]) [15]. Retrospective investigations of biomarkers related to the clinical trial were approved by the Regional Ethics Committee of Karolinska Institute, Stockholm, Sweden. Evaluations of estrogen receptor (ER) status, S-phase fraction (SPF), human epidermal growth factor receptor 2 (HER2) status, pAKT_S473 expression, mutations in *p53* and *PIK3CA, CDH1* gene copy number, fibroblast growth factor receptor 1 (*FGFR1*), and expression of fibroblast growth factor (*FGF3, FGF4*, and *FGF19*) mRNA have been described elsewhere [16-21].

### Colorectal cancer

Frozen material from primary colorectal tumors and corresponding histologically normal mucosa and from colorectal liver metastases were collected consecutively at Linköping University Hospital from 2006 to 2012 (approved by the Regional Ethical Review Board, Linköping, Sweden; file nos. M190-04 and M119-09, including written informed consent). To ensure optimal preservation of the RNA, tissue samples were placed in RNAlater (Applied Biosystems/Ambion Inc.) immediately after resection and stored at –70°C. Formalin-fixed paraffin-embedded (FFPE) tissues from primary and metastatic colorectal cancers (n=60) were collected at the Department of Pathology, Linköping University Hospital. For the liver metastases (n=36), the clinicopathological variables gender, age, tumor size, number of tumors, radical resection (R0/R1), synchronous/metachronous, extra hepatic disease and time to recurrence were analyzed. 

### Establishment of primary cell lines from colorectal tumors and liver metastases

Primary cell lines were established from primary tumors and liver metastases of patients undergoing surgery for colorectal cancer at Linköping University Hospital. Minced tumor tissue were cultured in RPMI 1640 (Fisher Scientific) supplemented with 20% fetal bovine serum (FBS) (Invitrogen), 10 mM HEPES (Invitrogen), 30 μg/mL gentamicin (Invitrogen), and 2.5 μg/mL Fungizone^®^ (Invitrogen). Cultures were cultivated at 37°C in 5% CO_2_. 

### MicroRNA and mRNA extraction from frozen and paraffin-embedded tissues

Total RNA was extracted from frozen tissue using a mirVana mirNA Isolation Kit (Applied Biosystems/Ambion) according to the manufacturer’s protocol. The concentration, purity, and integrity of RNA were determined using Nanodrop (Thermo Scientific) and an Agilent 2100 Bioanalyzer with an RNA 6000 Nano Chip Kit (Agilent Technologies Inc.). The microRNA and mRNA were extracted from paraffin-embedded tissues using a mirNeasy FFPE kit (Qiagene).

### Quantitative PCR of microRNAs and mRNAs

Expression of mir-200s, *ZEB1*, and *CDH1* mRNA were analyzed by quantitative RT-PCR (RT-qPCR). Unless not specified, all methods were performed according to the protocols provided by the manufacturer. TaqMan microRNA assays (Applied Biosystems) were used for hsa-mir-141, mir-200a, mir-200b, mir-200c, and mir-429. MicroRNA samples were analyzed in triplicates using RNU48 and r18S (Applied Biosystems) as endogenous controls. 

RNA was transcribed to cDNA using a High Capacity cDNA Reverse Transcription Kit (Applied Biosystems). The expression levels of *CDH1* and *ZEB1* were determined by TaqMan Gene Expression Assays (Applied Biosystems). mRNA samples were analyzed in triplicates, and as internal controls expression of 18S rRNA and PPIA (Applied Biosystems) was used in assays of the colorectal samples, and expression of ACTB and PPIA (Applied Biosystems) in assays of the breast cancer samples. All samples were analyzed on a 7900 Fast Real-Time PCR System (Applied Biosystems). Data obtained from the RT-qPCR was analyzed by the Comperative C_t_ method, normalized to the mean of the endogenous controls and the results are presented as relative expressions. For pairwise comparisons, matched normal mucosa from the same patient as the tumor material was used. 

### In situ hybridization

The localization of microRNA expression was determined by microRNA *in situ* hybridization (ISH) using a locked nucleic acid (LNA) probe for mir-141 and control probes for mir-205, nuclear U6, and scrambled microRNA, together with a MicroRNA ISH kit for FFPE tissues (Exiqon). This was performed according to manufacturer’s protocol using the parameters indicated below. Briefly, FFPE slides were deparaffinized, treated with proteinase-K (15 μg/mL) for 10 min, and then incubated with microRNA probe (50 nM) for 1 h at 56°C. For visualization, anti-DIG block solution with alkaline phosphatase (AP) antibody (1:800) (Roche Diagnostics) supplemented with goat serum (Jackson Immunoresearch) and NBT-BCIP tablets (Roche Diagnostics) was used.

### Microdissection

The differences in microRNA expression between matched epithelial tissues from normal colorectal mucosa, primary tumors, and liver metastases were verified by RT-qPCR analysis after tumor specific microdissection. Laser-capture microdissection (LCM) was performed using an Arcturus kit (Applied Biosystems), and total RNA was extracted and RT-qPCR was performed as described above.

### Immunohistochemistry staining

To stain for the E-cadherin protein, FFPE tissue sections were processed by deparaffinization in a PT-link rinse station followed by antigen retrieval for 20 min at 96°C (DakoCytomation). Samples were incubated at 4°C overnight with a rabbit monoclonal antibody (mAb) against human E-cadherin (24E10, Cell Signaling Technology) diluted 1:100 in TBST with 5% normal goat serum. The anti-rabbit Envision+ system conjugated with horseradish peroxidase (DakoCytomation) was used as a secondary reagent. The color was developed using 3.3-diaminobenzidin hydrochloride (DAB)/H_2_O_2_, and cell nuclei were counterstained with hematoxylin. Vimentin staining was performed at the Department of Pathology, Linköping University Hospital, according to routine procedures using an anti-vimentin mAb (clone RV202, BD Diagnostics) diluted 1:200 in an automated immunostainer (Biocare IntelliPATH Flex). 

### Statistical analysis

For the survival analyses, a combination variable, mir-200, was created and defined as high expression (upper quartile) of mir-141 and/or mir-200c. Where indicated, the continuous variables for *ZEB1*, *CDH1*, and microRNA expression were divided into four levels based on the quartiles. In the analyses of secondary metastatic recurrence from colorectal cancer, definitions of high and low expression were based on the median because of the limited number of samples. Spearman’s rank order correlation was used to determine the association between EMT factors and various markers of cancer signaling. The relationships identified between the different variables were assessed by Students t-test and the paired t-test, whereas for grouped variables we used the χ^2^ test, χ^2^ for trend, or Fisher’s exact test when appropriate. The product-limit method was employed to estimate cumulative probabilities of local recurrence-free survival (LRFS) and distant recurrence-free survival (DRFS). Differences in survival between groups were assessed by the log-rank test. Univariate and multivariate analyses of event rates were performed using Cox proportional hazard regression, which was also applied to analyze interactions between the different factors and treatments by including the variables X (potential predictive factor) and treatment, and the interaction variable (X * treatment). All the procedures were comprised in STATISTICA, version 9.0 (StatSoft, Inc. 2009). The criterion for statistical significance was *p* < 0.05.

## Results

### EMT factors associated in primary colorectal tumors also show associations in liver metastases and are related to progressive disease

**Figure 1:**
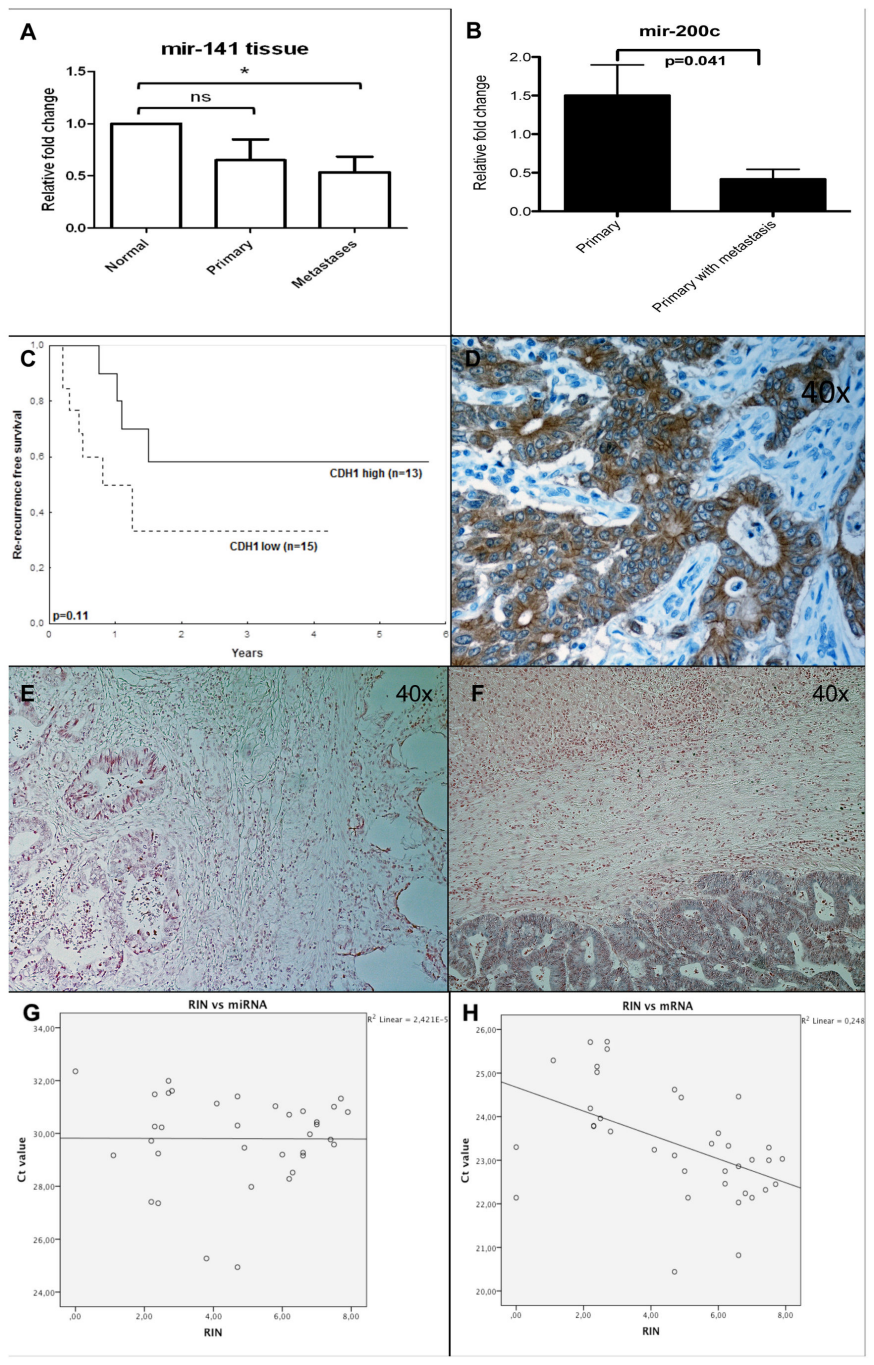
EMT factors in different compartments of clinical samples. In matched human colorectal cancer samples, the levels of mir-141 were significantly lower in metastatic tissue than in normal tissue (*p*=0.043) (*a*). Expression of mir-200s in primary colorectal tumors with known metastatic status revealed a significant association with metastatic disease for low levels of mir-200c, relative to matched normal mucosa (*p*=0.041) (*b*) and in the metastatic lesions low levels of CDH1 indicated a trend of shorter time to recurrence (*p*=0.11) (*c*). E-cadherin was expressed specifically in epithelial colorectal cancer cells (*d*). Mir-141 showed strongest expression in epithelial cells of the liver metastases as compared to normal tumor-adjacent mucosa and primary colorectal tumor (*e*, *f*). Staining of the probe was negative in stromal cells and in hepatocytes. The integrity of frozen tissue samples from cancer patients was assayed using a bioanalyzer. The Ct values of microRNAs were not correlated with the corresponding RNA integrity numbers (RIN) values (*g*), whereas the Ct values of mRNA were associated with the RIN values (*h*).

To study the role of genetic changes in EMT-related factors during cancer progression, matched human tissue samples were analyzed. Results regarding normal epithelial mucosa, primary colorectal tumor, and liver metastases derived from the same patient showed that mir-141 levels were significantly lower in metastatic tissues compared to normal tissues (*p* = 0.043, Figure 1a). Analysis of the expression of mir-200 family in primary colorectal tumors with known metastatic status showed that low levels of mir-200c were significantly associated with a metastatic disease (*p* = 0.041; Figure 1b).

To evaluate the role of EMT factors in metastatic lesions and to determine whether the expressions of these factors exhibit the same associations in secondary tumors as seen in colorectal and other primary tumors [9,10], colorectal liver metastases were analyzed. Expression levels of all the five members of the mir-200 family, *ZEB1*, and *CDH1* were measured by RT-qPCR in frozen tissue samples, and the results showed that the relationships between these factors in metastatic tissues were comparable those observed in primary tumors. The mir-200s were positively correlated with *CDH1* and negatively associated with *ZEB1* (Table 1). There was an inverse correlation between *CDH1* and *ZEB1* (R = –0.482, *p* = 0.001). 

**Table 1:** EMT signalling pathways are associated to colorectal liver metastases.

	**mir-200a**	**mir-200b**	**mir-200c**	**mir-141**	**mir-429**
**Tumor size** (<5, ≥5 cm)	R=0.46 *p*=0.007	R=0.37 *p*=0.03	R=-0.02 *p*=0.91	R=0.38 *p*=0.03	R=0.33 *p*=0.06
**Nr of tumors** (<3, ≥3)	R=-0.37 *p*=0.03	R=-0.37 *p*=0.04	R=-0.29 *p*=0.09	R=-0.33 *p*=0.07	R=-0.37 *p*=0.03
***CDH1***	R=0.53 *p*=0.002	R=0.63 *p*<0.0001	R=0.43 *p*=0.02	R=0.55 *p*=0.001	R=0.63 *p*<0.0001
***ZEB1***	R=-0.25 *p*=0.15	R=-0.35 *p*=0.06	R=-0.19 *p*=0.31	R=-0.37 p=0.04	R=-0.47 *p*=0.007

Clinicopathological characteristics and EMT pathway-related gene expression in correlation with microRNA 200 family member expressions in colorectal liver metastases.

It is known that the prognosis for individuals is worse when there is a high number of metastatic lesions in the liver (>3) compared to fewer metastases, but also that large tumors, (>5 cm) imply a worse prognosis than smaller tumors [22]. High expression levels of EMT factors correlated with large tumors but were negatively correlated with the number of metastatic lesions (Table 1). High number of tumors was associated with shorter survival whereas large tumors indicated a lower risk of liver metastatic recurrence (Figure S1a and b). A trend towards shorter time to progression was seen in patients with low levels of *CDH1* (*p*=0.11; Figure 1c) as well as members of the mir-200a/mir-200b/mir-429 cluster (*p*=0.22, *p*=0.08 and *p*=0.10, data not shown). Other clinicopathological variables investigated showed no significant associations (data not shown).

### MicroRNAs may be studied in clinical tissue samples but stromal tissue contribution affects the analyses of EMT factors

To investigate the potential of EMT factors as clinical markers for cancer progression and determine their cellular distribution, *in situ* hybridization (ISH) and immunohistochemistry were performed on FFPE tissues, collected and prepared during routine clinical procedures. E-cadherin protein was shown to be selectively expressed in epithelial cells of colorectal tumors (Figure 1d). In the epithelial cells of normal mucosa and primary and metastatic colorectal samples ISH revealed mir-141 to be specifically expressed, but no detectable differences in the level of expression between the stages of progression were found in these matched samples, although there was a trend towards higher expression in the metastatic leasion (Figure 1e and f). Expression of this microRNA was not detected in tumor stroma, and it should be noted that the size of the stromal compartment in both primary colorectal tumors and metastases varies between patients (Figure S2). 

To evaluate the stability of microRNAs in clinical tissue samples, the integrity of extracted material was assessed in frozen samples of liver metastases, derived from patients with colorectal cancer. The RNA integrity number (RIN) values, which ranged from undetectable to 9.5, were compared with cycle threshold (Ct) values obtained for microRNAs and mRNAs by RT-qPCR analysis (Figure 1g and h). The Ct values for the microRNAs were not dependent on the RIN values, which confirmed that microRNAs are stable in frozen tumor material, whereas mRNAs were found to be more prone to degradation. 

Taken together, these results show that EMT factors should be studied in the epithelial compartments, and microRNAs are stable and can be detected by ISH in FFPE tissue samples prepared by routine methods at pathology laboratories, and thereby could be useful as clinical markers.

### EMT plasiticity is implied in microdissected epithelial compartments of progressive colorectal cancer in matched patient tissue samples and confirmed in primary cultures of the same samples

**Figure 2:**
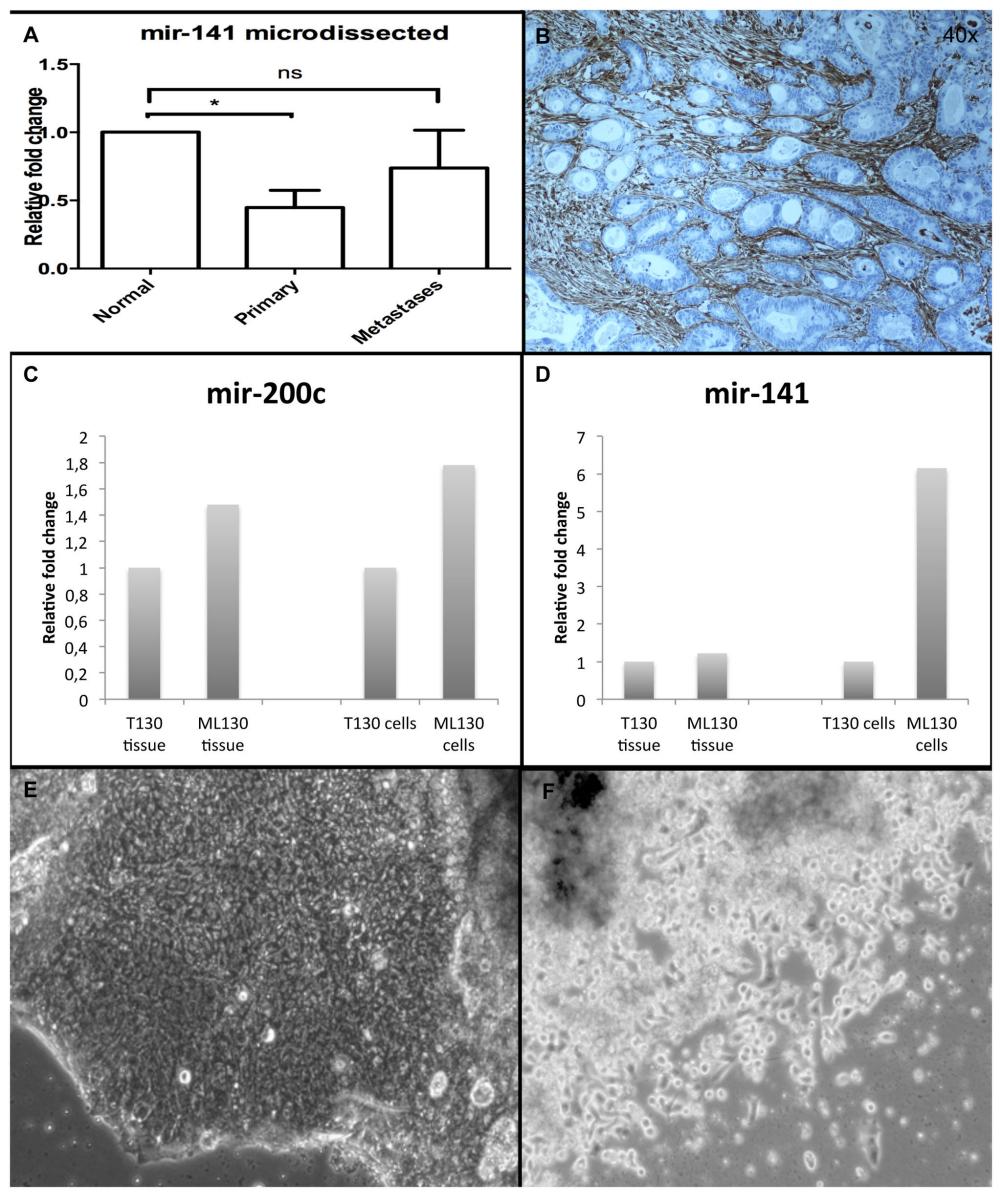
Metastatic tumors show altered mir-200 expression. Matched FFPE tissue samples from patients with metastatic colorectal cancer were microdissected (*a*). Compared to normal epithelial cells, primary colorectal cancer cells, but not metastatic cells, showed a significant decrease in mir-141 expression. Vimentin selectively stain the stromal compartment of primary and metastatic tumors (*b*). Measurement of mir-141 and mir-200c levels in primary cell cultures from matched primary colorectal cancer and liver metastases from the same patient confirmed an association between the two microRNAs equivalent to that detected in the microdissected tissue samples (*c*-*f*).

To selectively analyze the expression of mir-200 in the epithelial compartment, matched normal, primary, and metastatic FFPE tissue samples from patients with metastatic colorectal cancer were microdissected (Figure 2a). Samples of normal mucosal epithelial cells, primary colorectal tumor cells, and cells from colorectal liver metastases were analyzed by RT-qPCR in the same manner as described above. This showed a significant decrease in mir-141 in primary colorectal cancer cells (*p* = 0.016), but not in metastatic cells, as compared to the normal epithelial cells. These results were verified in the same samples analyzed by microRNA arrays (Applied Biosystems) (unpublished data). Vimentin expression was shown to be selectively expressed in stromal compartments of the tumors (Figure 2b), and in epithelial bile duct cells in the liver metastases (Figure S3). 

To further investigate EMT factors in the clinical samples, tumor cells were cultured from matched primary colorectal tumors and liver metastases, removed simultaneously from the same patient during surgery. The levels of mir-141 and mir-200c were measured and confirmed to be related in the same way as observed in the microdissected tissue samples from the same patient (Figure 2c–f). 

To summarize, in a complex multi-cell environment such as that present in tumors our findings demonstrate that it is essential to verify the methods applied and to use reliable study material.

### EMT factors in primary breast tumors

The evaluation of the EMT was extended by using a larger material consisting of breast tumors from 98 postmenopausal women with stage II disease. Since mir-141/200c had proven to be of greatest clinical relevance in the analyses of metastatic colorectal cancer samples, this microRNA cluster was further focused on in the analyses of the breast cancer material. 

The mean follow-up of the breast cancer cohort was 11 years, and data was available for a number of clinicopathological factors. The patients had been randomized to adjuvant treatment with radiotherapy or CMF, and thus it was also possible to correlate the results with treatment responses. For validation, the expression of the *CDH1* gene was compared with data on copy number variation for this gene that was previously obtained by assessment of Affymetrix 250K Nsp arrays in 29 of the tumors. This showed that there was a positive correlation between *CDH1* mRNA expression and gene copy number (R = 0.49, *p* = 0.027). In addition, both of the mir-200s in the analyzed cluster were positively correlated (R = 0.37, *p* = 0.00015). High expression of either member of the indicated mir-200 cluster was associated with substantial expression of *CDH1* mRNA (R = 0.22, *p* = 0.034), whereas *ZEB1* and *CDH1* mRNA were negatively correlated (R = –0.23, *p* = 0.027). 

### Mir-200s can serve as predictors of the response to radiotherapy and chemotherapy in breast cancer patients

**Figure 3:**
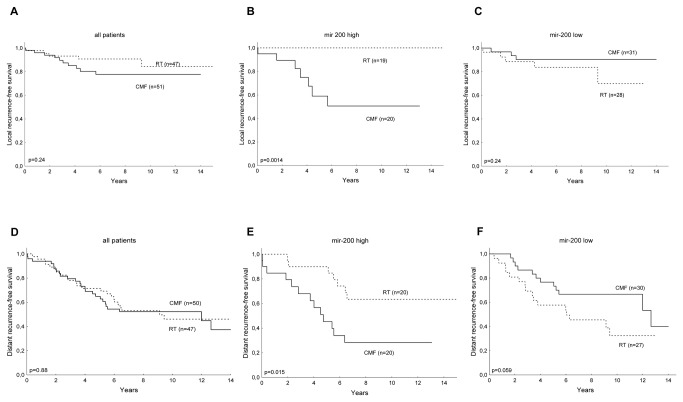
EMT-related factors act as treatment predictors in primary breast tumors. The treatment predictive value of mir-200 was analyzed in tumors from women who had been randomized to treatment with radiotherapy (RT) or systemic chemotherapy (CMF: cyclophosphamide, methotrexate, 5-FU) (*a*, *d*). After stratifying for mir-200 expression, RT reduced the risk of local recurrences in the group of patients with tumors expressing high levels of mir-200s (*b*) but not in the group of tumors showing low mir-200 levels (*c*). The difference in the risk of distant recurrence in relation to treatment (*e*, *f*) was significant (test for interaction *p* = 0.0035).

The outcome of breast cancer disease in connection to mir-200 expression in primary tumors was highly dependent on the adjuvant therapy regimen used. Radiotherapy significantly decreased the risk of local recurrence in the patients with tumors expressing high levels of mir-200 but not in the patients whose tumors had low mir-200 levels (Figure 3a, b and *c*). Conversely, low mir-200 expression was associated with pronounced benefits of CMF, chiefly with regard to distant recurrence-free survival (Figure 3d *, e* and *f*). The interaction between the treatment effect and miR-200 was clearly significant (CMF vs. radiotherapy: mir-200 *high* HR = 3.3 [CI 95% 1.3–9.42; *p* = 0.013] and mir-200 *low* HR = 0.49 [CI 95% 0.24–1.03; *p* = 0.059]; test for interaction *p* = 0.0035). This interaction was still significant in a multifactorial analysis including tamoxifen treatment, lymph node status, tumor size, ER status, HER2 status, SPF and these factors interactions with treatment effect (*p* = 0.00054).

To summarize, it is possible that this difference in the responses is related to the EMT, such that radiotherapy has the best effect on EMT-negative tumors, whereas systemic chemotherapy, targeting circulating tumor cells and micrometastases, is beneficial in cases exhibiting features of EMT. 

### The EMT-related mir-200 cluster has prognostic value in breast cancer patients

**Figure 4:**
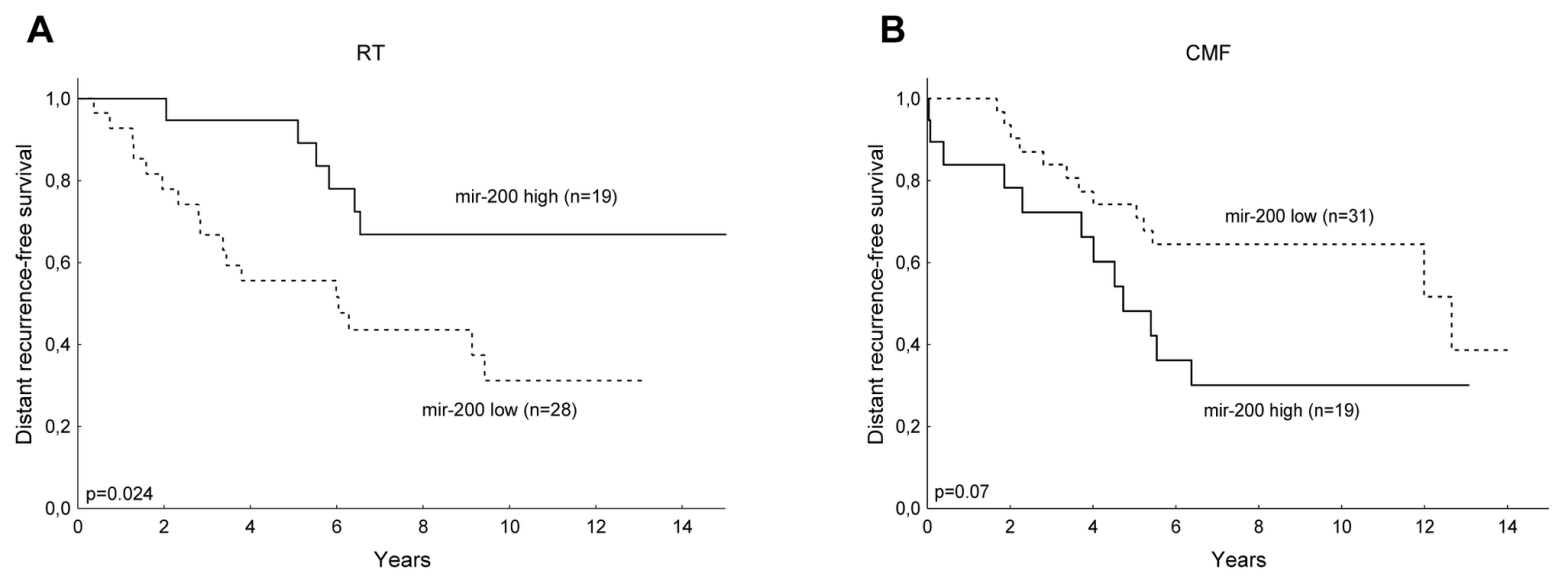
Prognostic value of EMT-related factors in primary breast tumors. In the group of breast cancer patients that did not receive chemotherapy, low expression of mir-141/200c was associated with significantly worse outcome in terms of distant recurrence-free survival (HR = 2.74, [CI 95% 1.08–6.99, *p* = 0.034]) (*a*). The opposite trend was seen for patients who received systemic chemotherapy (HR = 2.09, [CI 95% 0.95–4.58], *p* = 0.067) (*b*).

In the group of breast cancer patients that did not receive chemotherapy, low expression of mir-141/200c was associated with significantly worse outcome in terms of distant recurrence-free survival (HR = 2.74, [CI 95% 1.08–6.99], *p* = 0.034) (Figure 4a). The results were also significant in a multifactorial analysis including tamoxifen treatment, lymph node status, tumor size, ER status, HER2 status, and SPF (HR = 3.69, [CI 95% 1.11–12.2], *p* = 0.033). The opposite trend was seen for patients who received systemic chemotherapy (HR = 2.09, [CI 95% 0.95–4.58], *p* = 0.067) (Figure 4b). Considering clinicopathological variables, mir-200c was negatively correlated with lymph node status (R = –0.24, *p* = 0.0047), and mir-141 was negatively correlated with S-phase fraction (R = –0.22, *p* = 0.035). Together, these findings suggest that the EMT is involved in malignant progression and thus might be useful as a prognostic factor and a target for treatment of recurrent disease. 

### Associations between EMT and well-established factors involved in breast cancer signaling pathways

**Figure 5:**
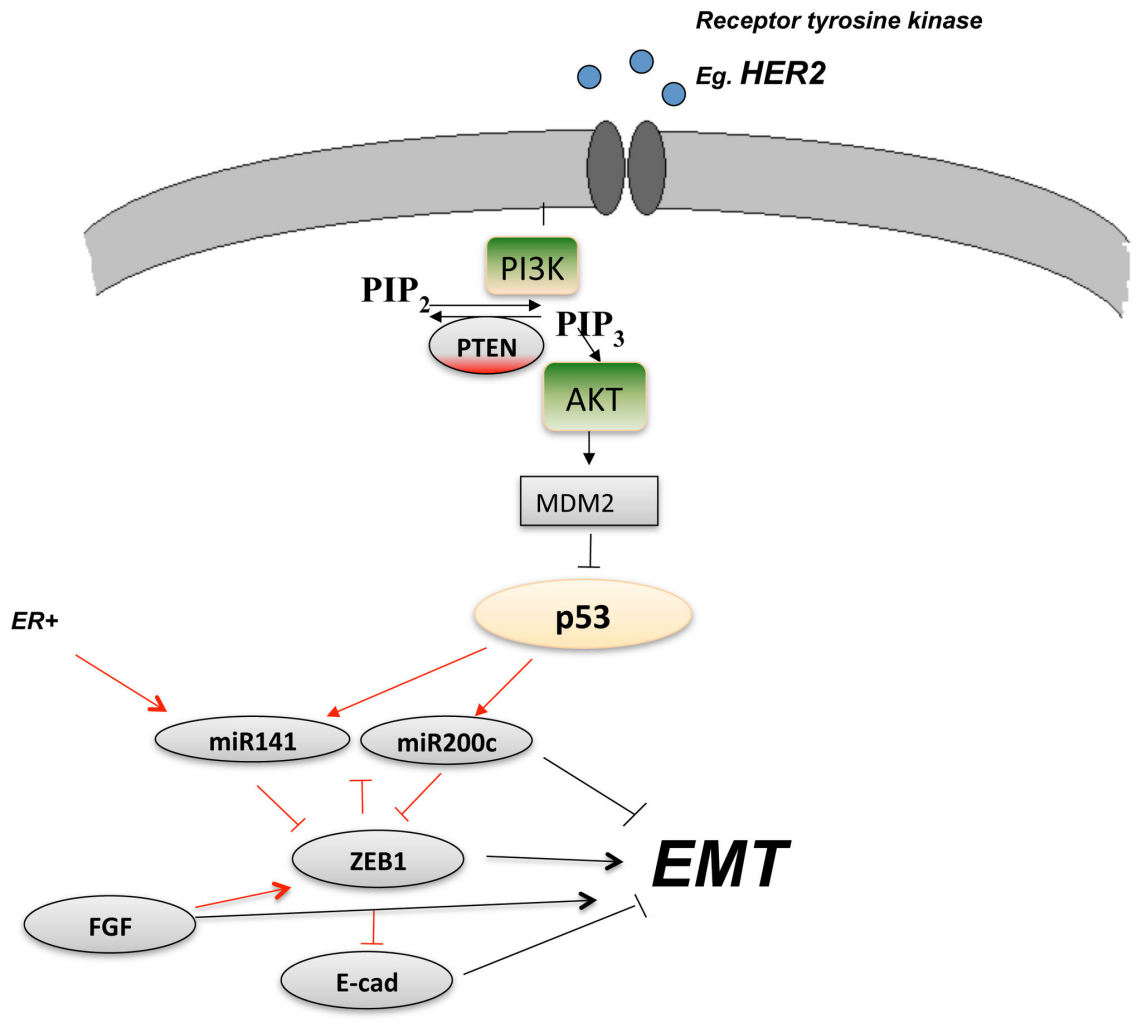
Associations between EMT and factors in cancer signaling pathways. Expression of EMT-related factors was associated with components of cancer signaling pathways (significant associations found in this study indicated in red).

In the breast cancer cohort, it is plausible that the expression of EMT markers is related to the PI3K–AKT–p53 signaling pathway (Figure 5). In support of that assumption, we found a negative correlation between mutated p53 and high expression of mir-141 (R = –0.26, *p* = 0.012) and mir-200c (R = –0.21, *p* = 0.039), which agrees with recently reported data suggesting that p53 is a negative regulator of the EMT pathway [23]. Furthermore, there was a positive correlation between mutated *PIK3CA* status and *ZEB1* (R = 0.30, *p* = 0.0042), whereas there was a negative correlation between *PIK3CA* mutations and expression of mir-141/mir-200c (R = –0.23, *p* = 0.032). A more in-depth evaluation of the group of patients with non-mutated p53 revealed a positive correlation between *ZEB1* and activated (phosphorylated) AKT (pAKT_S473) (R = 0.25, *p* = 0.045), although there was a tendency towards a negative correlation between these two variables in the p53-mutated group (R = –0.33, *p* = 0.13). Interestingly, mir-141 was positively correlated with ER status (R = 0.31, *p* = 0.0019), whereas mir-200c was negatively correlated with HER2 status (R = –0.25, *p* = 0.013), suggesting that these microRNAs play disparate roles in different subtypes of breast cancer. Furthermore, we considered alternative signaling routes in cancer plasticity and spread, which revealed interesting associations between expression of *ZEB1* and three genes involved in fibroblast growth factor signaling: *FGF4* (R = 0.34, *p* = 0.04), *FGF19* (R = 0.43 *p* = 0.0084), and *FGFR1* (R = 0.28, *p* = 0.092). These factors may contribute to the development of a mesenchymal phenotype. In summary, high *ZEB1*/low mir-200 expression was associated with activation of the PI3K–AKT pathway and with FGF signaling.

## Discussion

It has become clear that cancer research should be focused on metastatic disease, since this state represents the main cause of cancer-related deaths. The present study has provided translational findings concerning the role of microRNAs that regulate the EMT in colorectal and breast cancer. We investigated the potential of microRNAs as clinical markers and also evaluated the possibility of implementing those markers as new and rapid tools to complement the currently used routine methods at pathology departments. Our results show that EMT-associated factors such as *ZEB1* and E-cadherin, as well as microRNAs that regulate these factors, change during tumor progression. Additionally, we found that the levels of expression of EMT markers were significantly correlated with clinical outcome and response to treatment in colorectal and breast cancer. 

Resection of secondary tumors has become increasingly common practice in colorectal cancer, because experience has shown that this approach prolongs survival and offers the only chance of cure to patients with metastatic disease [24]. However, it is not yet possible to predict whether the patients will be cured by a particular treatment or have one or several recurrences that require multiple surgical strategies and/or radiation/chemotherapy approaches to halt disease progression. Unfortunately, markers for recurrent disease are lacking, and our knowledge of the crucial steps leading to metastasis is limited, strongly demonstrating that greater research efforts are needed in this field. Our studies on liver metastases reveal a negative association between size and number of metastases. We also show for the first time that the expression of the mir-200 family was associated with larger but fewer metastases, indicating a better prognosis for these patients. The shorter survival among patients with many tumors and higher risk of liver metastatic recurrence in patients with small tumors further support this theory. Moreover, small tumors were not significantly associated with shorter survival and many tumors did not predict liver metastatic recurrence, indicating local versus systemic spread. 

The reason that data on EMT and metastasis are inconclusive might be linked to the limited availability of reliable clinical samples and the fact that *in vitro* systems are inadequate and frequently have drawbacks related to the choice of cell lines and experimental interventions applied to the cells. It is also possible that the importance of EMT has been overestimated in the numerous reviews and *in vitro* studies on this topic, and thus it is obvious that EMT need to be investigated using human tissues. To elucidate the roles of various factors in cancer progression it is essential to study tissue samples of metastatic disease, and this can be best achieved by analyzing matched primary and metastatic tumors from the same patient. Indeed, such analyses have become more common [25,26], because tissue specimens collected at surgery are available to a greater extent today. Nevertheless, large cohorts are rare, and therefore it is preferable to use matched samples to ensure reliable data. We extended this strategy by including tumor-adjacent normal colorectal mucosa in our evaluation of normal epithelial cells and metastatic cells with the aim of determining the direction of changes in markers. 

Data from *in vitro* studies suggests that the EMT is involved in metastatic processes such as phenotypic transdifferentiation, cell movement, migration, and invasion [6,27,28]. In comparison, data obtained *in vivo* are in general occasional; although Xue and colleagues have convincingly shown that epithelial cells have different EMT statuses in mouse models of breast cancer [7,8]. In addition, Brabletz and coworkers [29,30] have demonstrated that cells in invasive areas of human tumor samples exhibit altered expression of various EMT markers (e.g., E-cadherin and ZEB), and Hur et al. [25] recently described the existence of EMT features in a cohort of colorectal cancer patients from Japan. Notably, it seems that the findings reported by Hur and colleagues are similar to what we observed in our Caucasian cohort, which is particularly interesting, considering that research results regarding colorectal cancer genetics often differ between different ethnic populations [31]. 

One explanation for the contradictory data on EMT in the literature may be disparities in the methodologies used in the different studies, for instance with respect to the choice of cell lines, preparation of samples, and tissue heterogeneity. Thus our aim was to use different methods to verify the validity of the results, and to ensure close collaboration between the laboratory, surgery, and pathology professionals in order to obtain reliable data derived from valuable patient materials.

When comparing gene expressions in tissues from different organs, it is important to consider the cellular composition of the samples, that is, the proportions of normal and malignant cells versus stromal cells. In many cases, it is impossible to determine whether the level of a factor of interest depends solely on the malignancy or is instead dictated by the tissue composition (e.g., established by stromal “dilution”). To clarify this issue, we performed microdissection of normal and malignant epithelial cells from samples of colorectal cancer tissue, and indeed received a different result compared to whole tissue samples from the same patients. Much of the literature on metastasis presents comparisons of whole tumor tissue samples from different organs [25,26,32]. Clearly, more comparable results should be achieved by comparing samples taken from the same type of tissue, although even in such a case it is necessary to have knowledge of the expression of the factors of interest (here EMT-related features) in different cell types. To verify this assumption, we performed immunohistochemistry and ISH on different kinds of tissues, and as well on purified and cultured primary cells from the same variety of tissues. Interestingly, the results for matched microdissected colorectal primary tumors and liver metastases were similar to those obtained for cultured primary cells from the same compartments. 

In our breast cancer samples, we found that low expression of the mir-141/mir-200c cluster was associated with activated signaling via the PI3K–AKT pathway, which concurs with recent data suggesting that p53 negatively regulates the ZEB–E-cadherin pathway by upregulating mir-200s [23]. We also observed that mir-141 was correlated with positive ER status, whereas mir-200c was negatively associated with HER2 status, which suggests that these microRNAs play different roles in different subtypes of breast cancer. 

FGF signaling and ZEB factors may contribute to cancer plasticity in diverse ways. However, our results do not indicate whether these two factors affect each other, but rather merely show that they are correlated, and other investigators have suggested that they may instead contribute to the EMT along different pathways [33].

Low expression of the mir-141/200c cluster was associated with a decrease in distant recurrence-free survival, which implies that these EMT markers can be useful prognostic factors in cancer. Our data support the theory that loss of expression of mir-200 family members activates the EMT system, and could thereby lead to migration and spreading of cells, as has been reported in various types of cancers [27,28,34]. Moreover, Davalos et al. [35] have published very detailed data on methylation of the mir-200s in microdissected human colon cancer that agree with our results. However, the findings of a breast cancer study conducted by Korpal and coworkers [26] indicated the opposite, and those investigators proposed that the underlying mechanism was mesenchymal-epithelial transition (MET) signaling; it should be noted that nothing was mentioned about the treatment used in the patient cohort in that investigation. Our results demonstrate that the indicated EMT markers can be useful treatment predictive factors in cancer, as mir-141/200c can serve as a predictor of response to radiation or chemotherapy in tumor progression. A potential explanation for the good response to radiotherapy in the presence of high levels of mir-200s is that such conditions allow local treatment to induce greater effect on adherent tumors. On the other hand, low levels of mir-200s may reflect an EMT-positive tumor with a more mesenchymal and migrating phenotype that can be addressed more efficiently by systemic treatment (e.g., with a chemotherapy regimen). It is plausible that EMT-positive cells can be targeted as circulating tumor cells, but to assess that possibility it would have been necessary to use a cohort of patients where also blood is sampled. Soluble factors, such as circulating cells or microvesicles, is getting increased focus as metastatic biomarkers and growing evidence indicate them to be informative of the metastatic process [36,37].

Conclusively, this data show the importance of taking into account the therapy given to the patients in the studied cohort when studying the prognostic value of EMT, and furthermore the different cell types in the specimens analyzed. Additionally, it may be important to examine numerous separate subsets or areas of the tumors to determine tumor heterogeneity. Although the use of tissue microarrays can provide large amounts of data, that approach also severs the evaluation of invasive areas, which may explain why some researchers have reported non-prognostic EMT data [10,38].

Notwithstanding, it is very convenient to be able to study whole tissue samples, because use of such material facilitates implementation of the procedure in the routine work performed at pathology laboratories. In our cohort of breast cancer tumors, even though we used unselected areas (i.e., any part) of whole tissue samples, we were able to discern relationships with clinical data and prognosis. However, it is impossible to know whether those associations were due to stromal dilution rather than to a decrease in levels of the mir-200s. 

To summarize, we have shown that the EMT-related mir-200–ZEB–E-cadherin signaling pathway is of clinical relevance in predicting metastatic progression of primary tumors and the responses to different treatments. Furthermore, microRNAs are relatively stable in tissue samples, and hence these biomolecules hold potential as novel clinical markers. Nevertheless, further work must be done to elucidate these results in relation to other findings. It is also plausible that additional microRNAs and signaling systems can play important roles in the metastatic process *in vivo*, perhaps in synergy with mir-200–ZEB. Unfortunately, it seems that levels of EMT factors fluctuate during metastatic progression, and thus they may not represent the most appropriate targets for treatment. Accordingly, it would be interesting to search for further factors that change in one direction along with the disease and therefore may be more suitable in a clinical context. 

## Supporting Information

Figure S1 **Prognostic value of tumor characteristics in colorectal liver metastases.** High number of metastatic lesions (>3) were associated with shorter survival (*a*), and large tumors (>5 cm) indicated lower risk of metastatic recurrence (*b*).
(TIF)

Figure S2 **In situ hybridization to microRNA in clinical samples.** Signals were detected using positive control probes: the small nuclear RNA U6 (*a*) and mir-205 (*b*). Omitting the probes or performing the analysis using scrambled microRNAs gave negative results (*c*, *d*). According to the Sanger microRNA database, brain tissue shows very little or no expression of mir-141, and thus we used such tissue as a negative control (NC) and confirmed that no ISH signal was present (*e*).
(TIF)

Figure S3 **Vimentin positive epithelial cell in colorectal liver metastases.** A few colorectal cancer samples did show a limited number of vimentin-positive epithelial cells, surrounded by stromal cells in the border between tumor and normal liver tissue but further investigation of those cells by use of antibodies against CK7 and CDX2 led the pathologist (HO) to conclude that they were proliferative bile duct cells.
(TIF)
