# Supplementary figures and images for: The Role of MicroRNA-200 in Progression of Human Colorectal and Breast Cancer
Source: PLoS One. 2013 Dec 20;8(12):e84815. doi: 10.1371/journal.pone.0084815 (PMC3869924; doi:10.1371/journal.pone.0084815)

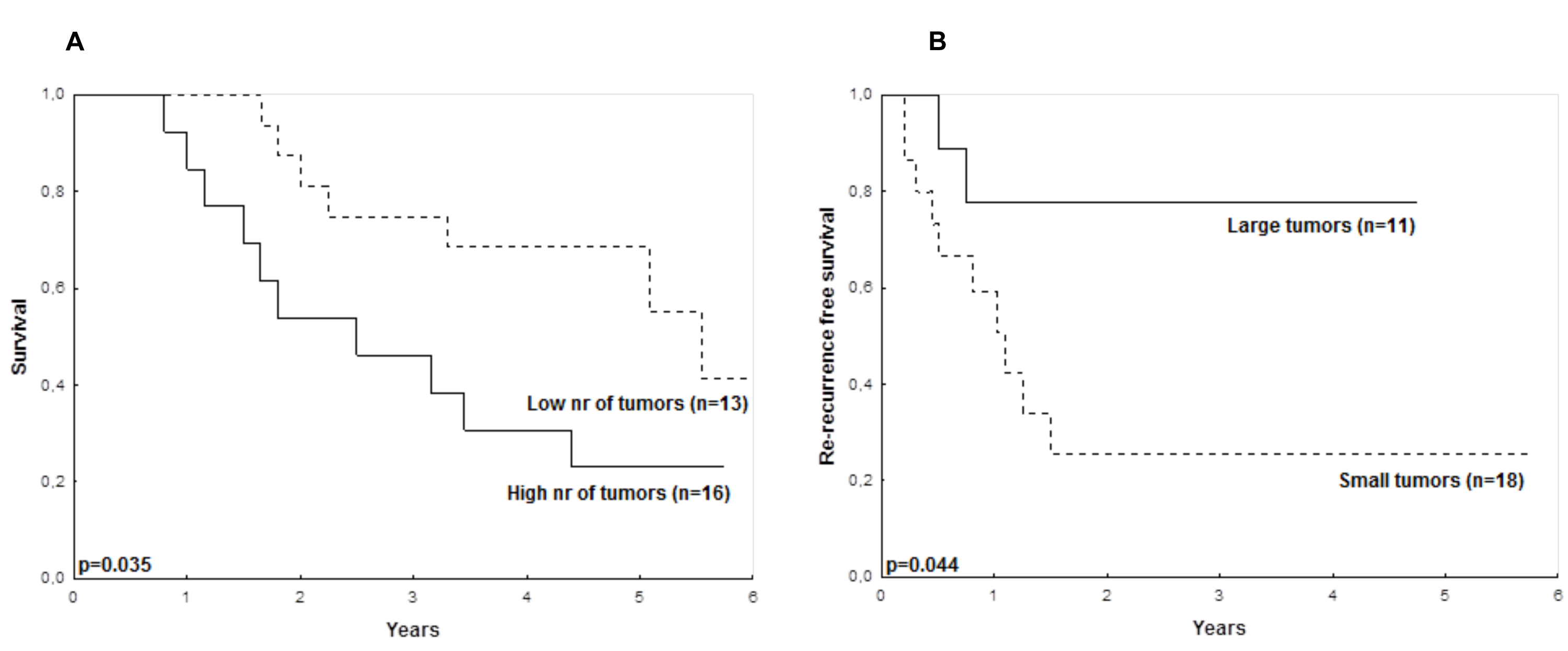

Supplement: Figure S1 — Prognostic value of tumor characteristics in colorectal liver metastases. High number of metastatic lesions (>3) were associated with shorter survival (a), and large tumors (>5 cm) indicated lower risk of metastatic recurrence (b). (TIF) [file pone.0084815.s001.tif]

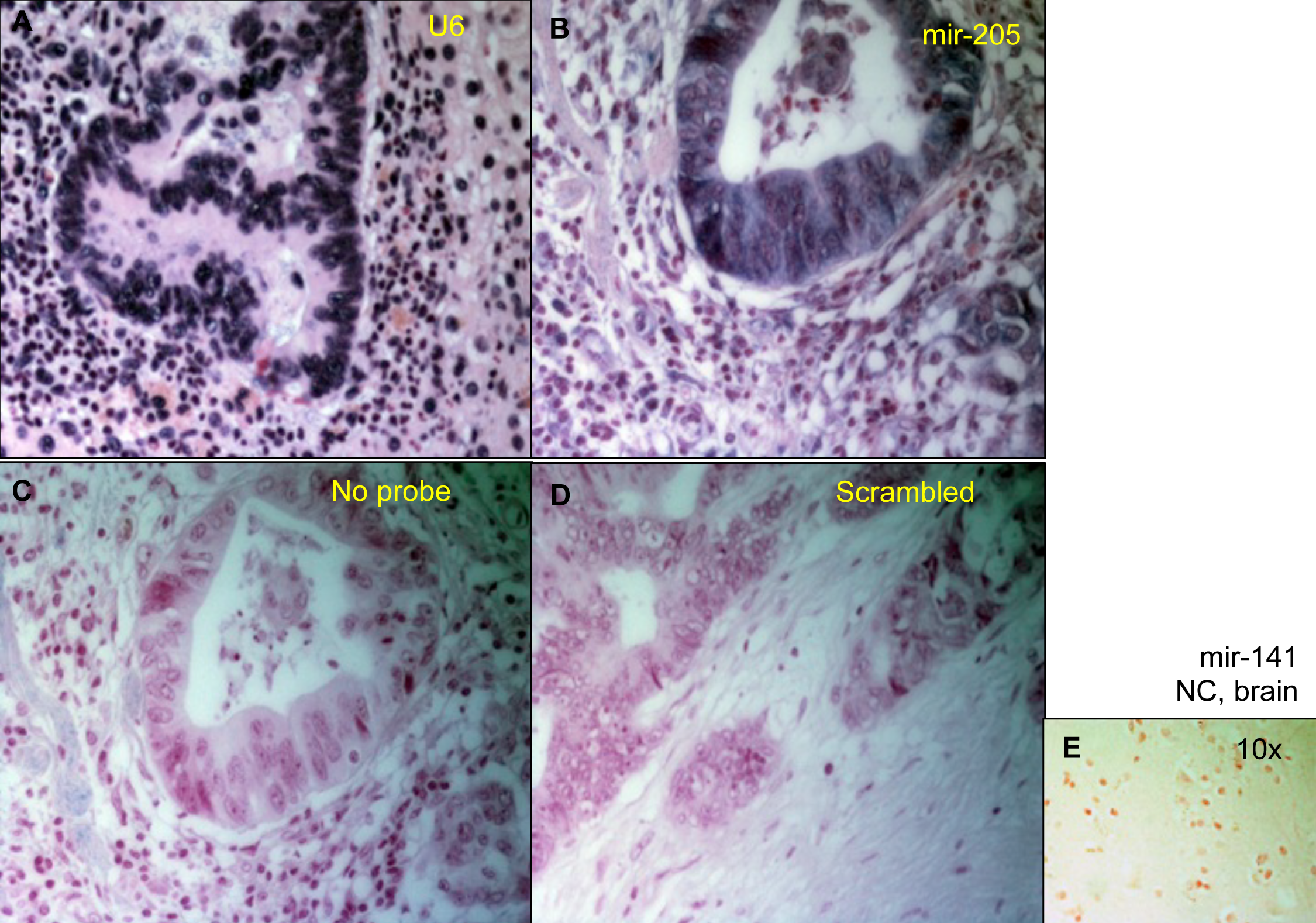

Supplement: Figure S2 — In situ hybridization to microRNA in clinical samples. Signals were detected using positive control probes: the small nuclear RNA U6 (a) and mir-205 (b). Omitting the probes or performing the analysis using scrambled microRNAs gave negative results (c, d). According to the Sanger microRNA database, brain tissue shows very little or no expression of mir-141, and thus we used such tissue as a negative control (NC) and confirmed that no ISH signal was present (e). (TIF) [file pone.0084815.s002.tif]

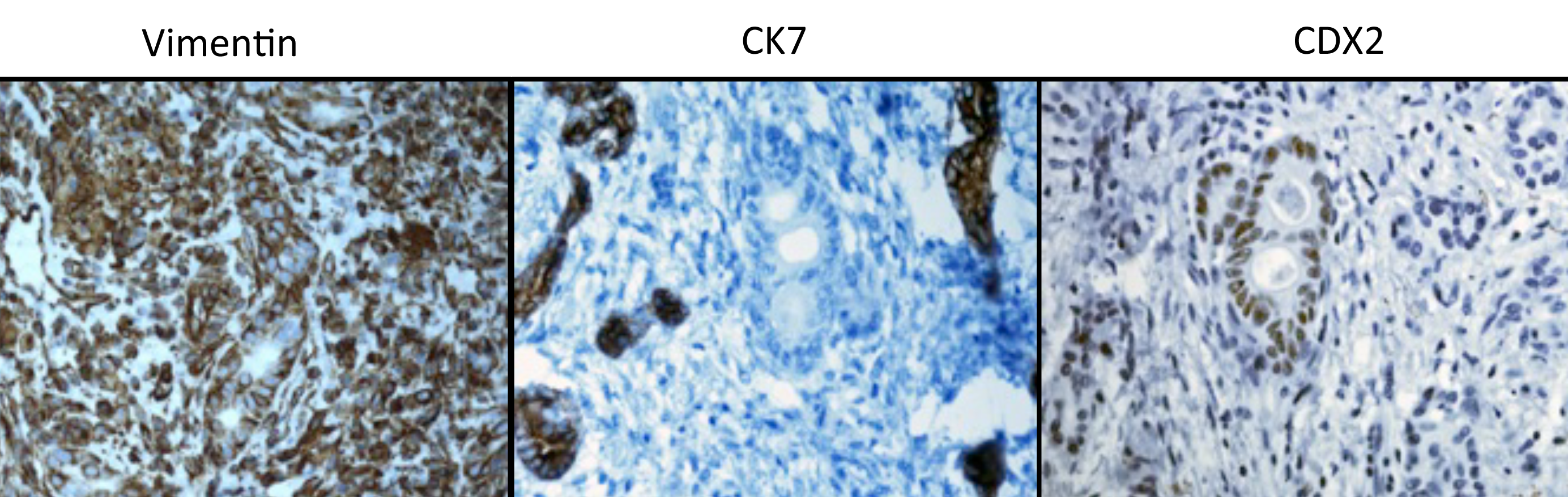

Supplement: Figure S3 — Vimentin positive epithelial cell in colorectal liver metastases. A few colorectal cancer samples did show a limited number of vimentin-positive epithelial cells, surrounded by stromal cells in the border between tumor and normal liver tissue but further investigation of those cells by use of antibodies against CK7 and CDX2 led the pathologist (HO) to conclude that they were proliferative bile duct cells. (TIF) [file pone.0084815.s003.tif]
